# Sex Differences in Spironolactone and the Active Metabolite Canrenone Concentrations and Adherence

**DOI:** 10.3390/biomedicines10010137

**Published:** 2022-01-08

**Authors:** Laura E. J. Peeters, Leonardien K. Tjong, Wim J. R. Rietdijk, Teun van Gelder, Birgit C. P. Koch, Jorie Versmissen

**Affiliations:** 1 Department of Hospital Pharmacy, Erasmus MC, University Medical Centre Rotterdam, 3015CN Rotterdam, The Netherlands; kimskie97@gmail.com (L.K.T.); w.rietdijk@erasmusmc.nl (W.J.R.R.); T.van_Gelder1@lumc.nl (T.v.G.); B.koch@erasmusmc.nl (B.C.P.K.); j.versmissen@erasmusmc.nl (J.V.); 2 Department of Internal Medicine, Erasmus MC, University Medical Centre Rotterdam, 3015CN Rotterdam, The Netherlands

**Keywords:** hypertension, sex differences, adherence, drug monitoring, spironolactone

## Abstract

We aim to investigate sex differences in blood concentrations of spironolactone and the active metabolite canrenone in resistant hypertension patients. Furthermore, sex differences in adherence for spironolactone and other antihypertensive drugs (AHDs) were studied. The patients in this post hoc study had all participated in a single-blind randomized controlled trial called RHYME-RCT (Dutch Trial Register, NL6736). Concentrations in blood of several AHDs were assessed in RHYME-RCT to investigate adherence to treatment. This allowed for a comparison of drug exposure to spironolactone and canrenone between males and females. In linear regression models, no statistically significant sex differences (N = 35) in spironolactone (B =−10.23, SE = 7.92, *p* = 0.206) or canrenone (B = 1.24, SE = 10.96, *p* = 0.911) concentrations after adjustment for dose and time between sampling and intake were found. Furthermore, no statistically significant differences in non-adherence to spironolactone were found between sexes (N = 54, male 15% vs. female 38%, *p* = 0.100), but non-adherence to spironolactone was associated with non-adherence to other AHDs (*p* ≤ 0.001). Spironolactone and canrenone concentrations were not different between males and females with resistant hypertension. Although not statistically significant, females were twice as likely to be non-adherent to spironolactone compared to males, and thereby also more likely to be non-adherent to other AHDs.

## 1. Introduction

Optimal pharmacological treatment is an important contributor in the management of hypertension to decrease the risk of cardiovascular diseases and death [1]. To achieve optimal treatment, numerous antihypertensive drugs are available. Choices are in part made based on comorbidities and clinical effects [1]. However, around 12–15% of all patients diagnosed with hypertension do not reach their blood pressure goals and, depending on the drug class, 3–7% of the patients discontinue antihypertensive drug therapy due to adverse events [2,3] Therefore, other factors that have an influence on the variation in response to antihypertensive drug use should be taken into account.

Recent research has indicated that males and females react differently to antihypertensive drug use due to differences in pharmacokinetics (PK) and pharmacodynamics (PD). This could potentially explain part of the variation in response to antihypertension drugs, which makes sex an interesting target to investigate and improve pharmacological treatment in patient with hypertension [4,5,6,7].

Differences in body composition and organ function between the sexes may particularly influence the PK of antihypertensive drugs. In general, the volume of distribution, basal metabolic clearance, and renal clearance is higher in males as compared to females. This can, for some antihypertensive drugs, lead to an increased drug exposure in females as compared to males when receiving the same drug dose. In turn, a higher exposure in the body could potentially lead to a stronger effect on blood pressure, but also to more adverse events. A study of Rydberg et al. reported a higher prevalence of adverse events in women after the use antihypertensive drugs, which increased when several antihypertensives were used concomitantly [8]. Moreover, both the incidence of adverse events and the number of prescribed antihypertensive drugs were associated with an increase in non-adherence and as a consequence to treatment failure [9,10].

However, all mentioned aspects contributing to sex differences in antihypertensive treatment response are largely based on assumptions, and extensive research on this topic is lacking. Only a few studies have measured antihypertensive drug concentrations in blood and compared these concentrations between males and females [4,5,11]. In a previous study, we showed that females had higher blood concentrations of canrenone, the active metabolite of spironolactone, after correction of the dose, time between intake and sampling, age, and body mass index (BMI). As this study was not designed for the purpose of finding sex differences, we wanted to confirm these findings in a second population. This second population had to consist of a more specific patient population while aldosterone antagonists, such as spironolactone, are not first-line therapy in the treatment of hypertension. Therefore, we selected patients with resistant hypertension. These patients use three or more antihypertensive drugs from different drug classes and still have uncontrolled blood pressure. As blood pressure control is challenging in this population, more information on how to optimize therapy can be helpful [12].

We hypothesize that females have higher concentrations of the active metabolite canrenone as compared to males, which in turn leads to more non-adherence to spironolactone. As such, the purpose of our current study is to determine the differences in whole blood concentrations of spironolactone and canrenone between males and females in a population with apparent resistant hypertension. Next to the primary objective, this study will also examine whether females are more likely to be non-adherent to spironolactone as compared to males.

## 2. Materials and Methods

### 2.1. Participants

Patients were selected from a single-blinded randomized controlled trial called RHYME-RCT (Resistant Hypertension: MEasure to ReaCh Targets, Dutch trial register, NL6736) if they used spironolactone. For this trial, patients were included at the vascular medicine and nephrology outpatient clinics of eleven hospitals throughout the Netherlands including two tertiary hospitals.

All patients were ≥18 years and fulfilled the criteria of resistant hypertension, which was defined as having an office blood pressure of >140 and/or 90 mmHg despite a medication regime of at least three antihypertensive drugs including a diuretic or at least four antihypertensive drugs from different drugs classes in the most optimal dose. Patients were excluded if they had end-stage renal disease (eGFR < 15 mL/min), insufficient understanding of the Dutch or Turkish language, or if secondary forms of hypertension were not established yet. Written informed consent was signed by every patient before participation in the trial. The RHYME-RCT trial was approved by the local Medical Ethical Committee, Rotterdam, the Netherlands (MEC-2018-027).

### 2.2. Study Design

The trail consisted of four measurement visits and four visits to the outpatient clinic. Only measurements from the first measurement visit were used to determine sex differences in spironolactone drug levels. During this first visit, written informed consent was obtained, a blood pressure measurement was carried out, and dried blood spot (DBS) sampling was performed to determine antihypertensive drug concentrations in blood. The DBS sample was sent to the pharmacy laboratory for analysis with a validated ultra-high-performance liquid chromatography–tandem mass spectrometry (UPLC-MS/MS).

After DBS sampling, the time of sampling and time of intake of each antihypertensive drug was verified with the patient and noted if possible. These values were used to determine the interval between the time of drug intake and blood sampling.

### 2.3. Measurements

#### 2.3.1. Sampling Method

DBS is a novel sampling method to simultaneously qualify and quantify antihypertensive drugs by detecting the drug levels by means of UPLC-MS/MS. DBS was performed by means of a finger prick and a drop of blood that was sampled on a card with filter paper. The actual analysis of drug levels was performed by means of a validated UPLC-MS/MS method as previously published [13]. This method also included the active metabolite of spironolactone, called canrenone. This enables measurement over a longer time period, while spironolactone is rapidly metabolized after intake and thereby undetectable in blood after approximately 7 h [13,14]. In contrast, canrenone levels in blood can be measured for at least 24 h after intake [13].

#### 2.3.2. Adherence

When both spironolactone and canrenone levels were undetectable in blood, patients were identified as non-adherent. This definition was also the case to determine non-adherence to other antihypertensive drugs that patients used in addition to spironolactone. Patients were identified as partially adherent when at least one drug was detectible in blood, but one or more other antihypertensive drug was absent. When all measured antihypertensive drugs were detectable, the patient was categorized as adherent.

Patient characteristics, sex, age, weight, renal function, and comorbidities were collected for all included patients at inclusion. Moreover, dosages of spironolactone of all included patients were collected through the healthcare information system.

### 2.4. Statistical Analysis

We analyzed our data using the following steps. First, patient characteristics were determined for the total study population and the subgroups males and females, which were used to determine adherence differences. We also made an additional table to present characteristics of a subgroup of patients based on data available for the regression analysis. 

For continuous variables, we present the mean and standard deviation (SD) and median and interquartile range (IQR) to estimate the glomerular filtrate rate (eGFR) measured with Chronic Kidney Disease Epidemiology Collaboration (CKD-EPI) because a cut-off value of 90 mL/min was used to report this value. For the categorical variable, we present numbers (%). The normal distribution of all continuous variables was determined by means of a boxplot and Shapiro–Wilk test. Testing differences in continuous variables between females and males, we used unpaired *t*-tests for continuous variables that were normally distributed and the Mann–Whitney U-test for non-normally distributed variables. For categorical variables, we used Chi^2^ tests when frequencies were expected to be >5 and Fisher’s exact test when frequencies were expected to be <5.

Second, spironolactone and canrenone concentrations from every patient were visualized in a concentration–time curve. Furthermore, the influence of sex on spironolactone and canrenone concentrations was assessed by calculating and comparing the geometric means from men and females using linear regressions. We adjusted the linear regressions for the drug concentration with the spironolactone dose and the time interval between spironolactone ingestion and blood sampling with DBS. The estimates of the linear regression are betas and standard errors. In addition, for both females and males, the overall area under the curve (AUC) of canrenone was calculated with the trapezoid method. Concentrations of canrenone were normalized to a dose of 12.5 mg.

Third, we compared the number of adherent patients using spironolactone with those who were non-adherent in men as well as in females. Furthermore, we also used it to determine sex differences in adherence to the other antihypertensive drugs used, and it determined a possible relation between adherence to spironolactone with adherence to other used antihypertensive drugs. Given the retrospective nature of the study that is based on data from a randomized controlled trial (i.e., RHYME-RCT), no power calculation was performed. We performed all statistical analyses using SPSS (version 25.0.0.1 for Windows; IBM Corp. Amonk, NY, USA). For the figures, we used GraphPad Prism 9.1.2 software (GraphPad Software, La Jolla, CA, USA).

## 3. Results

### 3.1. Study Population

A total of 54 patients (33 (61.1%) males and 21 (38.9%) females) on spironolactone treatment were included in the present study. The mean age of the patient group was 58.2 ± 10.5 years (Table 1). No statistically significant difference was found in the dose of spironolactone between females (mean 39.3 mg, SD 24.1 mg) and males (mean 36.7 mg, SD 23.1 mg). Patients were on treatment with a mean of 4.6 antihypertensive drugs. No difference was found between sex and the use of the types of antihypertensive classes (Table S1). Besides creatinine, no relevant significant differences were observed between males and females in baseline characteristics (Table 1).

### 3.2. Difference in Spironolactone and Canrenone Concentrations between Sexes

**Figure 1:**
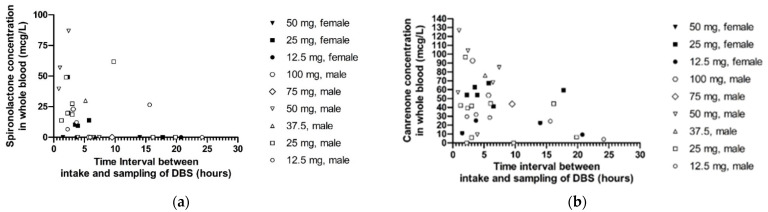
(**a**) Concentration–time curve of spironolactone. Open dots indicate spironolactone of males and closed dots (black) of females. Each dose (taken once daily) is represented by a different icon. (**b**) Concentration–time curve of canrenone, the active metabolite of spironolactone. Open dots indicate canrenone of males and closed dots (black) of females. Each dose (taken once daily) is represented by a different icon.

To determine the difference between sexes with regard to spironolactone and canrenone concentration, data on time of intake and sampling and dose had to be available. In some cases, the patient was non-adherent to spironolactone, resulting in the absence of detectable concentrations of spironolactone and the canrenone-metabolite in blood. From 35 out of the 54 patients, all relevant variables were present, of which 13 patients were excluded due to non-adherence and 6 patients due to missing variables such as the time of intake. Patient characteristics of this selection of patients (N = 35) are displayed in Table 2. An overview of all the measured concentrations of spironolactone as well as canrenone are shown in Figure 1. The following dosages were used by males: 12.5 mg OD (once daily) (N = 5), 25 mg OD (N = 10), 37.5 mg OD (N = 1), 50 mg OD (N = 6), 75 mg OD (N = 1), and 100 mg OD (N = 2). In comparison, females used the following dosages: 12.5 mg OD (N = 2), 25 mg OD (N = 6), and 50 mg OD (N = 2).

The linear regression analyses showed that sex had no significant influence on the spironolactone or canrenone concentration after correction for dose and time between intake and sampling (Table 3). This analysis also showed that concentrations of both compounds decreased over time. However, this was only statistically significant for spironolactone (*p* = 0.034). Furthermore, an increased dose of spironolactone was associated with an increase in canrenone concentrations in blood even after correction for time of intake and sex (*p* = 0.042) (Table 3). Other covariates such as age, BMI, and eGFR were separately tested in a linear regression model with dose and time between intake and sampling included but had no statistically significant effect on the canrenone or spironolactone concentrations. The calculated AUC of canrenone for males was 314.2 mcg/L/24 h, and for females, the AUC was 415.8 mcg/L/24 h.

### 3.3. Antihypertensive Drug Adherence between Sexes

From all the patients that used spironolactone (N = 54), 31.7% were non-adherent for spironolactone and 36.6% of the patients were non-adherent for at least one of the remaining antihypertensive drugs that were used. Females were twice more often non-adherent for spironolactone and the remaining antihypertensive drugs as compared to males (spironolactone female 38% vs. male 15%; other antihypertensive drugs female 43% vs. male 18%) (Table 4), but this was not statistically significant (*p* = 0.100 and *p* = 0.054). Nevertheless, a relationship was found between adherence to spironolactone and adherence to the remaining antihypertensive drugs (Table 4). Patients who were non-adherent for spironolactone were more likely to also be non-adherent for at least one of the remaining antihypertensive drugs patients used compared to those who are adherent for spironolactone (62% vs. 20%, *p* ≤ 0.001).

## 4. Discussion

This is the first study to investigate sex differences in spironolactone and canrenone concentrations by means of measuring drug levels in a population of resistant hypertension patients and simultaneously included data on adherence. This is a unique study as sex differences are often based on theoretical differences, and only a limited number of studies have measured drug concentrations in blood to determine actual differences. Additionally, most of these studies used healthy volunteers to establish differences to avoid factors that have an influence on drug concentrations [11,15]. By measuring in a real-life population, in this case patients with resistant hypertension, results can be translated more easily to clinical practice.

Our study demonstrated that measured concentrations of spironolactone and the active metabolite canrenone in whole blood were not significantly different in females compared to males. These findings contradict the results of a previous study of Peeters et al. [5]. However, it must be noted that the patient population differed in both studies. The previous study included patients with controlled blood pressures with two or more antihypertensive drugs, while the current study included resistant hypertension patients with uncontrolled blood pressures and three or more antihypertensive drugs. The inclusion of a different patient population has an influence on the results, firstly because patients with resistant hypertension have more comorbidities and take a wide range of antihypertensive drug classes (Table S1). This can influence the pharmacokinetics and dynamics of spironolactone. Secondly, an increased number of prescribed antihypertensive drugs is associated with increased non-adherence [9], resulting in more zero values of spironolactone concentrations in patients with resistant hypertension.

The choice to select patients with resistant hypertension resulted in differences in characteristics between males and females. A significant difference between the sexes occurred in the creatinine level, which is a measure for kidney function. This could be relevant as spironolactone metabolites are mainly excreted via the kidneys and thereby influence the concentrations in blood [16]. However, the difference in creatinine levels was to be expected as men have higher muscle mass and thereby higher creatinine levels compared to females. Furthermore, since the formula to calculate the CKD-EPI corrects for sex differences, the difference between males and females disappeared. Therefore, we assumed there were no sex differences in kidney function.

Besides the differences in patient population, other matrices were used to measure spironolactone concentrations. The current study used whole blood for measurement and analysis, while Peeters et al. [5] used blood plasma. Although the data from plasma and DBS cannot be directly compared in analysis, a difference between males and females in the same direction would be expected.

The study of Peeters et al. also showed that there was a large variability in drug concentrations of patients taking the same dose [5]. Despite this variability when taking the same dose, a higher dose almost always resulted in higher concentrations as compared to a lower dose. When reviewing the concentrations of spironolactone and canrenone, we found a few inconsistencies in the found concentrations on a certain time point in combination with the dose of spironolactone. An example of these inconsistencies can be found at Figure 1a, point 10 h, 25 mg, male e.g., 25 mg male at 10 h. A possible explanation is that some patients were not meticulous with the intake of spironolactone. This could explain why no relationship was found between the spironolactone dose and concentrations in the linear regression.

Previous studies have shown more non-adherence in females compared to males when using antihypertensive drugs [9,17]. This was in accordance with our results, wherein females were more than twice as likely to be non-adherent for either spironolactone or other antihypertensive drugs compared to males. However, no significant relationship between sex and adherence of spironolactone or sex and the remaining antihypertensive drugs was present in our analyses. A possible explanation for the higher non-adherence in females is a 1.2 times higher AUC of canrenone in females compared to males. These AUCs suggests that females are exposed to higher concentrations of spironolactone/canrenone after taking the same dose of spironolactone as males. These higher concentrations could have led to more side effects in females, resulting in more non-adherence, which was independently associated with lower adherence [18].

Patients who were non-adherent for spironolactone were more likely to be non-adherent for at least one of the remaining antihypertensive drugs they were using. This is in line with the previous observation that patients tend to be more non-adherent when the number of antihypertensive drugs they use increases. Following the guidelines, spironolactone is particularly used when triple therapy with other antihypertensive drugs does not work effectively [1]. Thus, it must be noted that the significant correlation between spironolactone adherence and other antihypertensive drugs adherence might be interchangeable: being non-adherent leads to the definition of resistant hypertension and prescription of spironolactone. It would be interesting to study adherence rates when spironolactone is used as monotherapy, for instance, for primary aldosteronism.

Our current study must be interpreted within the context of several possible limitations. First, we did not exclude concentrations below the lower limit of quantification, which was established at 31.2 µg/L for canrenone and 10.0 µg/L for spironolactone. Although this can lead to some deviation from the true value, these deviations will be small and will occur in both groups. Therefore, all results were included in the linear regression analysis.

Second, our sample size was smaller than expected, as many patients did not have all the necessary data that were needed for the analysis. Increasing the sample size for spironolactone analysis could in theory lead to a, probably small, significant difference, but it remains unclear whether this would also lead to a clinically relevant difference.

Third, only the total absence of detectable drug in blood was used to determine non-adherence. This does not exclude patients that are partially adherent and deviate from a once-daily regimen. Some measured concentrations will therefore be lower or higher than can be expected from the time after intake. The only solution to prevent for this is supervised intake, which was not part of the RHYME-RCT protocol. However, considering the long half-life of canrenone, the frequency of use should be very low to explain undetectable drug levels. Since no pharmacogenetic variants are known to influence spironolactone/canrenone levels, being a rapid metabolizer based on genotype such as is known for other drugs such as metoprolol cannot explain undetectable drug levels.

Lastly, the females included in our study were most often post-menopausal with a mean age of 57.5 years [19]. Consequently, hormonal changes must be taken into account; for instance, the renin-angiotensin-aldosterone system (RAAS) becomes more active after menopause [20,21,22]. This could also explain the, on average, slightly higher dose of spironolactone prescribed to females. Since it is still unknown how drug concentrations are influenced by hormonal changes, a larger study with stratification within females should be performed to draw conclusions for premenopausal women.

Furthermore, we do need to take into account that the results may not be extrapolated to all antihypertensive patients because our study population consisted of patients with resistant hypertension. Therefore, it would be interesting to include patients that are more similar to the general hypertension population. For instance, patients with primary hyperaldosteronism where spironolactone is the first choice in treatment. Additionally, a broader age distribution could show the influence of age on possible differences in concentration levels.

This analysis showed no clear relationship between sex and the concentrations of spironolactone and/or canrenone in resistant hypertension patients. This raises the question of whether there are any sex differences with regard to antihypertensive drugs that will lead to problems or therapy changes in clinical practice. With only a limited number of positive studies, mainly carried out in healthy volunteers, and no evident negative studies on this topic, this question remains unanswered for now.

## 5. Conclusions

Spironolactone and canrenone concentrations were not different between males and females with resistant hypertension. Although not statistically significant, females tend to be more non-adherent to spironolactone as compared to males, which might be due to a higher exposure, as suggested by higher AUCs, and thereby more side effects. This could also be an explanation as to why females are more likely to be non-adherent to other antihypertensive drugs.

## Figures and Tables

**Table 1:** Baseline characteristics patients from RHYME-RCT trial using spironolactone.

Characteristic	All (N = 54)	Female (N = 21)	Male (N = 33)	*p*-Value
Age, y, mean ± SD	58.2 ± 10.5	57.4 ± 10.7	58.8 ± 10.5	0.64
BMI, kg/m^2^, mean ± SD	30.9 ± 6.2	32.7 ± 7.4	29.8 ± 5.1	0.10
Creatinine, μmol/L, mean ± SD	96.2 ± 36.2	75.5 ± 21.3	109.4 ± 37.7	<0.01 *
CKD-EPI eGFR, mL/min/1.73 m^2^, median (IQR)	76.5 (61.0–87.5)	80.0 (64.5–90.0)	76.0 (53.5–86.5)	0.28
Used antihypertensive drugs, mean ± SD	4.6 ± 0.9	4.5 ± 1.0	4.6 ± 0.9	0.14
Spironolactone dose, mg, mean ± SD	37.7 ± 23.4	39.3 ± 24.1	36.7 ± 23.1	0.70
Comorbidity, N (%)				
Diabetes	16 (29.6)	4 (19.1)	12 (36.4)	0.18
Stroke	6 (11.1)	2 (9.5)	4 (12.1)	0.77
Coronary artery disease	7 (13.0)	3 (14.3)	4 (12.1)	0.82
Hypercholesterolemia	16 (29.6)	9 (42.9)	7 (21.2)	0.09
Heart failure	3 (5.6)	0 (0.0)	3 (9.1)	0.16
Asthma/COPD	3 (5.6)	2 (9.5)	1 (3.0)	0.32
Peripheral vascular disease	2 (3.7)	1 (4.8)	1 (3.0)	0.75
Aneurysm	1 (1.9)	0 (0.0)	1 (3.0)	0.43
Atrial fibrillation	2 (3.7)	1 (4.8)	1 (3.0)	0.75
None	16 (30.0)	6 (28.6)	10 (30.3)	0.89

* *p*-Value < 0.05 was found significant. SD: standard deviation, IQR: interquartile range, BMI: Body Mass Index (<18.5 = underweight, 18.5–24.9 = normal weight, 25–29.9 = overweight, 30–34.9 = obese, >35 = extremely obese), CKD-EPI eGFR: estimated glomerular filtration rate calculated with the Chronic Kidney Disease Epidemiology Collaboration formula.

**Table 2:** Baseline characteristics patients from RHYME-RCT trial used in linear regression analysis to determine sex differences in spironolactone and canrenone-metabolite concentrations.

Characteristic	All (N = 35)	Female (N = 10)	Male (N = 25)	*p*-Value
Age, y, mean ± SD	58.8 ± 11.4	60.3 ± 11.9	58.2 ± 11.4	0.64
BMI, kg/m^2^, mean ± SD	30.2 ± 5.6	30.2 ± 6.5	30.2 ± 5.3	0.99
Creatinine, μmol/L, mean ± SD	98.9 ± 36.7	73.8 ± 12.0	108.9 ± 38.6	<0.001 *
CKD-EPI eGFR, mL/min/1.73m^2^, median (IQR)	76.0 (62.0–84.0)	74.5 (65.8–82.3)	77.0 (53.0–86.5)	0.28
Prescribed antihypertensive drugs, mean ± SD	4.4 ± 0.9	4.5 ± 1.0	4.4 ± 0.9	0.77
Spironolactone dose, mg, mean ± SD	34.3 ± 22.3	27.5 ± 12.9	37.0 ± 24.9	0.15
Comorbidity, N (%)				
Diabetes	12 (34.3)	2 (20.0)	10 (40.0)	0.26
Stroke	4 (11.4)	1 (10.0)	3 (12.0)	0.87
Coronary artery disease	6 (17.1)	2 (20.0)	4 (16.0)	0.78
Hypercholesterolemia	11 (31.4)	5 (50.0)	6 (24.0)	0.13
Heart failure	3 (8.6)	0 (0.0)	3 (12.0)	0.25
Asthma/COPD	3 (8.6)	2 (20.0)	1 (4.0)	0.13
Peripheral vascular disease	1 (2.9)	1 (10.0)	0 (0.0)	0.11
Atrial fibrillation	2 (5.7)	1 (10.0)	1 (4.0)	0.49
None	10 (28.6)	3 (30.0)	7 (28.0)	0.91

* *p*-Value < 0.05 was found significant. SD: standard deviation, IQR: interquartile range, BMI: Body Mass Index (<18.5 = underweight, 18.5–24.9 = normal weight, 25–29.9 = overweight, 30–34.9 = obese, >35 = extremely obese), CKD-EPI eGFR: estimated glomerular filtration rate calculated with the Chronic Kidney Disease Epidemiology Collaboration formula.

**Table 3:** Linear regression analysis on the influence of covariates on spironolactone and canrenone concentrations.

	Spironolactone Whole Blood Concentration			Canrenone Whole Blood Concentration		
	B (SE B)	β	*p*-Value	B (SE B)	β	*p*-Value
Sex (ref = male)	−10.23 (7.92)	−0.214	0.206	1.24 (10.96)	0.133	0.911
Dose	−0.08 (0.17)	−0.076	0.656	0.49 (0.23)	0.343	0.042 *
Difference in time between intake and sampling DBS	−1.27 (0.58)	−0.369	0.034 *	−1.52 (0.80)	−0.304	0.065
N	35			35		
R square	0.106			0.181		
F-test	2.349		0.092	3.503		0.027 *

* Statistically significance at alpha-level of 5%; SE = standard error, DBS = dried blood spot.

**Table 4:** Adherence of spironolactone and other drugs between sexes.

Adherence Spironolactone	N (%)		
	Female (N = 21)	Male (N = 33)	*p*-Value
Adherent	13 (61.9)	28 (84.9)	0.100
Non-adherent	8 (38.1)	5 (15.2)	
Adherence Other *	**N (%)**		
	Female (N = 21)	Male (N = 33)	*p*-Value
Adherent	12 (57.1)	27 (81.8)	0.054
Partially adherent	4 (19.1)	5 (15.2)	
Non-adherent	5 (23.8)	1 (3.0)	
	**N (%)**		
Adherence Other *	**Adherence Spironolactone**		***p*-Value**
	Adherent	Non-adherent	≤0.001
Adherent	34 (82.3)	5 (38.5)	
Partially adherent	6 (14.6)	3 (23.1)	
Non-adherent	1 (2.4)	5 (38.5)	

*p*-Value < 0.05 was found significant. * Other: remaining antihypertensive drugs being used by patients (other than spironolactone).

## Data Availability

The data that support the findings of this study are available from the corresponding author, (L.E.J.P.), upon reasonable request.

## Supplementary Materials

The following are available online at https://www.mdpi.com/article/10.3390/biomedicines10010137/s1, Table S1: Percentages of used antihypertensive drug classes between females and males included in RHYME-RCT trial and used spironolactone.

## References

[1] Williams B., Mancia G., Spiering W., Rosei E.A., Azizi M., Burnier M., Clement D.L., Coca A., De Simone G., Dominiczak A. (2018). 2018 ESC/ESH Guidelines for the management of arterial hypertension: The Task Force for the management of arterial hypertension of the European Society of Cardiology (ESC) and the European Society of Hypertension (ESH). Eur. Heart J..

[2] Judd E., Calhoun D.A. (2014). Apparent and true resistant hypertension: Definition, prevalence and outcomes. J. Hum. Hypertens..

[3] Ross S.D., Akhras K.S., Zhang S., Rozinsky M., Nalysnyk L. (2001). Discontinuation of antihypertensive drugs due to adverse events: A systematic review and meta-analysis. Pharmacother. J. Hum. Pharmacol. Drug Ther..

[4] Eugene A.R. (2016). Metoprolol Dose Equivalence in Adult Men and Women Based on Gender Differences: Pharmacokinetic Modeling and Simulations. Med. Sci..

[5] Peeters L.E.J., Feyz L., Boersma E., Daemen J., van Gelder T., Koch B.C.P., Versmissen J. (2020). Clinical Applicability of Monitoring Antihypertensive Drug Levels in Blood. Hypertension.

[6] Di Giosia P., Giorgini P., Stamerra C.A., Petrarca M., Ferri C., Sahebkar A. (2018). Gender Differences in Epidemiology, Pathophysiology, and Treatment of Hypertension. Curr. Atheroscler. Rep..

[7] Ueno K., Sato H. (2011). Sex-related differences in pharmacokinetics and pharmacodynamics of anti-hypertensive drugs. Hypertens. Res..

[8] Rydberg D.M., Mejyr S., Loikas D., Schenck-Gustafsson K., von Euler M., Malmström R.E. (2018). Sex differences in spontaneous reports on adverse drug events for common antihypertensive drugs. Eur. J. Clin. Pharmacol..

[9] Gupta P., Patel P., Štrauch B., Lai F.Y., Akbarov A., Marešová V., White C.M., Petrak O., Gulsin G., Patel V. (2017). Risk Factors for Nonadherence to Antihypertensive Treatment. Hypertension.

[10] Gebreyohannes E.A., Bhagavathula A.S., Abebe T.B., Tefera Y.G., Abegaz T.M. (2019). Adverse effects and non-adherence to antihypertensive medications in University of Gondar Comprehensive Specialized Hospital. Clin. Hypertens..

[11] Cabaleiro T., Román M., Ochoa D., Talegón M., Prieto-Pérez R., Wojnicz A., López-Rodríguez R., Novalbos J., Abad-Santos F. (2012). Evaluation of the Relationship between Sex, Polymorphisms in CYP2C8 and CYP2C9, and Pharmacokinetics of Angiotensin Receptor Blockers. Drug Metab. Dispos..

[12] Oliveras A., De La Sierra A. (2013). Resistant hypertension: Patient characteristics, risk factors, co-morbidities and outcomes. J. Hum. Hypertens..

[13] Peeters L., Feyz L., Hameli E., Zwart T., Bahmany S., Daemen J., Van Gelder T., Versmissen J., Koch B.C.P. (2020). Clinical Validation of a Dried Blood Spot Assay for 8 Antihypertensive Drugs and 4 Active Metabolites. Ther. Drug Monit..

[14] Sica D.A. (2005). Pharmacokinetics and Pharmacodynamics of Mineralocorticoid Blocking Agents and their Effects on Potassium Homeostasis. Hear. Fail. Rev..

[15] Luzier A.B., Killian A., Wilton J.H., Wilson M.F., Forrest A., Kazierad D.J. (1999). Gender-related effects on metoprolol pharmacokinetics and pharmacodynamics in healthy volunteers. Clin. Pharmacol. Ther..

[16] Abshagen U., Rennekamp H., Luszpinski G. (1976). Pharmacokinetics of spironolactone in man. Naunyn-Schmiedebergs Arch. Fur Exp. Pathol. Pharmakol..

[17] Sportiello L., Rafaniello C., Sullo M.G., Nica M., Scavone C., Bernardi F.F., Colombo D.M., Rossi F. (2016). No substantial gender differences in suspected adverse reactions to ACE inhibitors and ARBs: Results from spontaneous reporting system in Campania Region. Expert Opin. Drug Saf..

[18] Tedla Y.G., Bautista L.E. (2016). Drug Side Effect Symptoms and Adherence to Antihypertensive Medication. Am. J. Hypertens..

[19] Gold E.B. (2011). The Timing of the Age at Which Natural Menopause Occurs. Obstet. Gynecol. Clin. North Am..

[20] Coylewright M., Reckelhoff J.F., Ouyang P. (2008). Menopause and hypertension: An age-old debate. Hypertension.

[21] Barton M., Meyer M.R. (2009). Postmenopausal hypertension: Mechanisms and therapy. Hypertension.

[22] Maas A.H.E.M., Franke H.R. (2009). Women’s health in menopause with a focus on hypertension. Neth. Hear. J..

