# Supplementary material for: Sex Differences in Spironolactone and the Active Metabolite Canrenone Concentrations and Adherence
Source: Biomedicines. 2022 Jan 8;10(1):137. doi: 10.3390/biomedicines10010137 (PMC8773364; doi:10.3390/biomedicines10010137)
Supplement: Supplementary file 1 [file biomedicines-10-00137-s001.zip › biomedicines-1461308-supplementary.pdf]

Supplementary Materials

Table S1. Percentages of used antihypertensive drug classes between females and males included in RHYME-RCT trial and used spironolactone.

	Female (N=21)		Male (N=33)		Chi^2-test
	**1 drug (N, (%))**	**>1 drug (N, (%))**	**1 drug (N, (%))**	**>1 drug (N, (%))**	***p*-Value**
ACE-inhibitors	7 (33.3)		9 (27.3)		0.63
ARBs	13 (61.9)		23 (69.7)		0.55
Beta blockers	14 (66.7)		24 (72.7)		0.63
Calcium-antagonists	16 (76.2)		31 (93.9)		0.06
Diuretics[¥]	9 (42.9)	10 (47.6)	17 (51.5)	13 (39.4)	0.82
Other[¥]	8 (38.1)	1 (4.8)	9 (27.3)	2 (6.1)	0.50

p-Value < 0.05 was found significant. ACE=Angiotensin-converting enzyme; ARB=Angiotensin II receptor blocker, RHYME-RCT=Resistant Hypertension: MEasure to Reach Targets. [¥]The diuretic and other group include a large number of different drug classes whereby the number of drugs can be more than 1.
